# Peritoneal dialysis outcomes in patients with nephrotic syndrome: a propensity score-matched cohort study

**DOI:** 10.1080/0886022X.2020.1792316

**Published:** 2020-07-20

**Authors:** Si-Jia Zhou, Ya-Kun Cong, Qing-Feng Han, Wen Tang, Tao Wang

**Affiliations:** a Department of Nephrology, Peking University Third Hospital, Beijing, China; b Department of Nephrology, The Forth Hospital of Daqing, Daqing, China

**Keywords:** Nephrotic syndrome, peritoneal dialysis, end-stage renal disease, outcomes

## Abstract

**Introduction:**

It is unclear whether patients with end-stage renal disease (ESRD) and nephrotic syndrome (NS) can be treated with peritoneal dialysis (PD).

**Objectives:**

To investigate the outcomes of PD treatment in ESRD patients with or without NS.

**Methods:**

In this retrospective cohort study, all incident patients with ESRD and NS who started PD from 1 February 2006 to 31 December 2017, were matched with patients without NS using propensity scores based on age, sex, diabetes mellitus status, and serum albumin.

**Results:**

Fifty-three patients in the NS PD group and 53 matched controls were included. The median survival of the NS PD group was comparable to that of the non-NS PD group. An interaction effect was observed between survival time and baseline NS status. Thus, patients’ outcomes within and after 1.5 years were analyzed separately. Both mortality (log-rank test, *p*= .235) and technique failure (log-rank test, *p*= .543) rates within 1.5 years in patients with NS were comparable to those of the non-NS group. After 1.5 years, however, the NS status at baseline was associated with lower all-cause mortality (*p*= .020) and lower technique failure (*p*= .008) rates in PD patients compared with the non-NS group. The multivariable Cox regression analysis showed that compared with the patients in the non-NS PD group, PD patients with NS had both significantly lower all-cause mortality and lower technique failure rate after adjusting for other factors.

**Conclusions:**

Our study indicates that PD may be considered as a long-term renal replacement therapy for patients with ESRD and baseline NS.

## Introduction

Nephrotic syndrome (NS) is characterized by heavy proteinuria with decreased serum albumin levels and is one of the best-known presentations of kidney disease in adults [1]. Nearly, 26% of patients with steroid-resistant NS younger than 20 years old developed end-stage renal disease (ESRD) in 5 years and need to receive long-term dialysis treatment. Peritoneal dialysis (PD) is an established renal replacement therapy in patients with ESRD. Although the use of dialysis is determined by both medical and nonmedical factors [2], evaluating whether differences exist in the mortality and technique outcomes of patients with and without NS on PD is of considerable interest.

In NS, generally, loss of urinary albumin cannot be compensated by increased albumin synthesis in the liver, and thus, hypoalbuminemia and hypoproteinemia are observed [3]. On PD, moreover, the amount of protein loss through the peritoneum is additive to albuminuria, leading to the concern of malnutrition [4,5]. Therefore, patients with NS and ESRD are usually thought to be not suitable to receive PD treatment. Previous reports on the efficiency of PD for NS and ESRD are relatively scarce. Most of them are case reports in children with refractory NS and overhydration [6–9]. For adult patients with NS, PD treatment reports are even fewer. Effective fluid removal without worsening the renal function has been discovered as an important merit of PD [10,11]. However, for patients with NS and ESRD, the long-term prognosis of PD therapy has not been fully studied and needs to be further elucidated.

In the present study, we retrospectively analyzed an incident cohort of PD patients with long-term follow-up and tried to determine whether patients with ESRD and NS treated with PD have adverse outcomes compared with those without NS. Accordingly, we hope to provide useful information for clinicians’ decision-making in modality choices for patients with ESRD and NS.

## Materials and methods

### Study design

**Figure 1.**
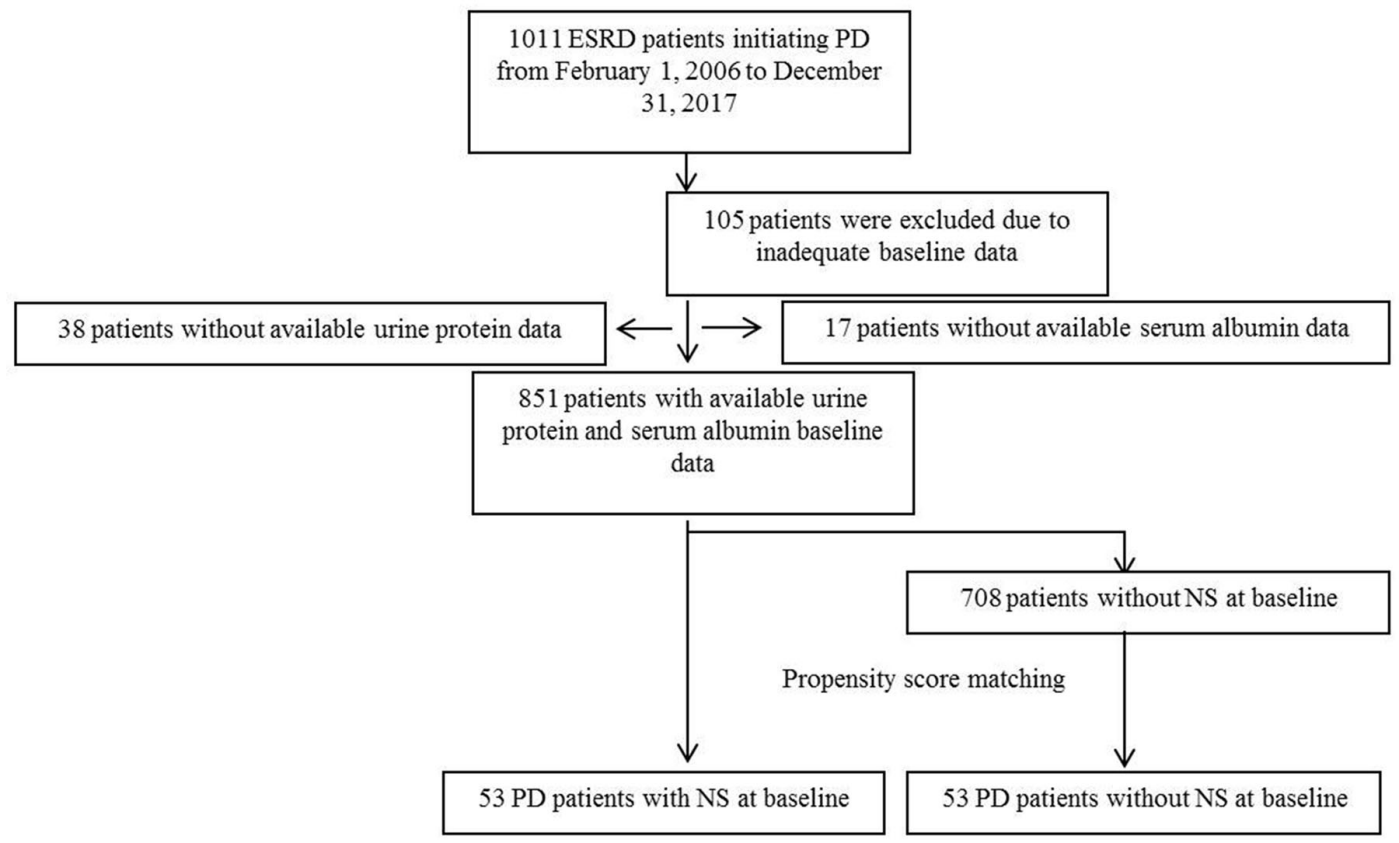
Flowchart of patient recruitment for this study.

This retrospective cohort study included all patients with ESRD who commenced PD between 1 February 2006 and 31 December 2017 in Peking University Third Hospital. In our center, patients need to be hospitalized to the department of nephrology for PD catheter implantation. Therefore, patients’ detailed kidney disease history as well as other disease histories was recorded on admission. During the study period, patients’ hospitalization medical charts were carefully reviewed to obtain the data on kidney disease as well as the evidence for the diagnosis of NS. A flowchart of patient recruitment for this study is shown in Figure 1. A total of 1011 incident PD patients were screened. Among these patients, 105 patients without sufficient baseline information, 38 patients whose proteinuria values were not available, and 17 patients without baseline blood albumin data were excluded. Therefore, 851 patients with ESRD aged more than 18 years with available longitudinal clinical information were selected. Among all these patients, 53 patients who met the diagnostic criteria of NS, that is, 24-h urine protein level greater than 3.5 g and blood plasma albumin level less than 30 g/L, were defined as the NS PD group. These patients were matched with control patients without NS by propensity score (PS) matching. The PS was calculated using multivariable logistic regression, in which NS was the outcome variable and age, sex, diabetes, and plasma albumin were the independent variables. The derived PSs were then used to match patients with NS with controls in a 1:1 ratio. Thus, a total of 106 patients (53 patients in the NS PD group and 53 matched controls in the non-NS PD group) were recruited in the final survival analyses. All patients were followed until 1 March 2018. This study was approved by the Peking University Third Hospital ethical committee (IRB00006761-M2019153).

### Endpoints

Participants were prospectively followed since the day they initiated PD treatment until death, renal transplantation, transfer to other centers, technique failure, or the end of study. The primary outcomes were all-cause mortality on PD (censored for loss to follow-up, renal transplantation, recovery of the renal function, transferring to hemodialysis (HD), and end of study) and technique failure (censored for loss to follow-up, renal transplantation, and end of study). Technique failure was defined as the transition of ESRD therapeutic modality from PD to HD permanently or death.

### Statistical analyses

The results are expressed as frequencies and percentages for categorical variables, mean ± standard deviation for continuous normally distributed variables, and median (interquartile range) for continuous variables that were not normally distributed. Continuous normally distributed data were compared using two-tailed unpaired *t*-tests. Continuous non-normally distributed data were compared using the Mann–Whitney tests. Dichotomous and categorical data were compared using the chi-square tests. PS matching was performed using Stata/SE version 12.0 (StataCorp, College Station, TX). Time-to-event analysis was evaluated using the Kaplan–Meier and Cox proportional hazards survival analyses. Statistical analysis was performed using IBM SPSS Statistics software (version 22.0, Armonk, NY) and Stata/SE version 12.0 (StataCorp, College Station, TX). *p* Values less than .05 were considered statistically significant.

## Results

### Patient demographic data

All patients were followed until 1 March 2018. A total of 53 patients in the NS PD group and 53 matched controls in the non-NS PD group were included. The median follow-up time was 3.32 (0.84, 5.95) years in the present study. At the end of follow-up, death occurred in 53 (50.0%) patients. A total of 18 patients (17.0%) transferred to HD. Four patients (3.8%) received renal transplantation and one (0.9%) had recovery of the renal function during the follow-up. Loss to follow-up occurred in two patients (1.9%), and six patients (5.7%) transferred to other dialysis centers.

The baseline comparison between patients in the NS PD and non-NS PD groups is depicted in Table 1. After PS matching, patients in the two groups showed no significant difference in matched variables (age, sex, and diabetes mellitus) except serum albumin at baseline, possibly due to the substantially lower serum albumin levels of patients with NS than those of the non-NS PD group even after matching. Moreover, there was no significant difference in systolic and diastolic blood pressure, height, weight, body mass index (BMI), hemoglobin, serum creatinine, estimated glomerular filtration rate, serum calcium, serum phosphate, serum parathyroid hormone, incidence of coronary heart disease, stroke, and hypertension between the NS PD and non-NS PD groups.

**Table 1.** Clinical parameters of the study population after propensity score matching at baseline.

Characteristics	NS PD group (*n* = 53)	Non-NS PD group (*n* = 53)	*p-* value
Age (years)	56.8 ± 14.6	57.8 ± 16.3	.746
Gender (male, %)	38 (72%)	40 (75%)	.659
Medical history			
Diabetes mellitus	33 (62%)	31 (58%)	.691
Coronary heart disease	4 (8%)	10 (19%)	.085
Stroke	8 (15%)	7 (13%)	.780
Hypertension	50 (94%)	49 (92%)	1.000
SBP (mmHg)	156.9 ± 27.2	149.3 ± 18.0	.109
DBP (mmHg)	88.6 ± 13.3	86.6 ± 15.7	.492
Height (cm)	167.4 ± 7.2	168.1 ± 7.0	.616
Weight (kg)	70.3 ± 11.7	65.9 ± 13.2	.095
BMI (kg/m^2^)	24.9 ± 3.7	23.4 ± 3.7	.062
Hemoglobin (g/L)	86.8 ± 19.2	83.1 ± 19.2	.332
Serum creatinine (μmol/L)	620.0 (458.5, 822.0)	670.0 (513.5, 964.5)	.175
Serum urea (mmol/L)	24.8 ± 7.8	26.2 ± 9.4	.412
eGFR (mL/min/1.73 m^2^)	7.7 (5.2, 10.5)	6.9 (4.2, 8.9)	.152
Serum albumin (g/L)	26.5 ± 3.2	28.0 ± 2.5	.007
Serum calcium (mmol/L)	1.9 ± 0.2	1.9 ± 0.3	.576
Serum phosphorous (mmol/L)	1.8 ± 0.5	1.8 ± 0.6	.921
PTH (pg/mL)	171.6 (81.1, 291.0)	168.0 (100.2, 314.6)	.926
24-h urine protein (g)	6.41 (4.71, 8.80)	1.49 (0.92, 3.13)	<.001
TCHO (mmol/L)	5.31 ± 1.77	4.59 ± 1.52	.032
TG (mmol/L)	1.63 ± 0.77	1.56 ± 0.95	.683
HDL-C (mmol/L)	1.16 ± 0.52	1.10 ± 0.36	.524
LDL-C (mmol/L)	3.30 ± 1.37	2.67 ± 1.27	.019

NS: nephrotic syndrome; PD: peritoneal dialysis; SBP: systolic blood pressure; DBP: diastolic blood pressure; BMI: body mass index; eGFR: estimated glomerular filtration rate; PTH: parathyroid hormone; TCHO: total cholesterol; TG: triglyceride; HDL-C: high density lipoprotein cholesterol; LDL-C: low density lipoprotein cholesterol.

### All-cause mortality of incident PD patients with or without NS

**Figure 2.**
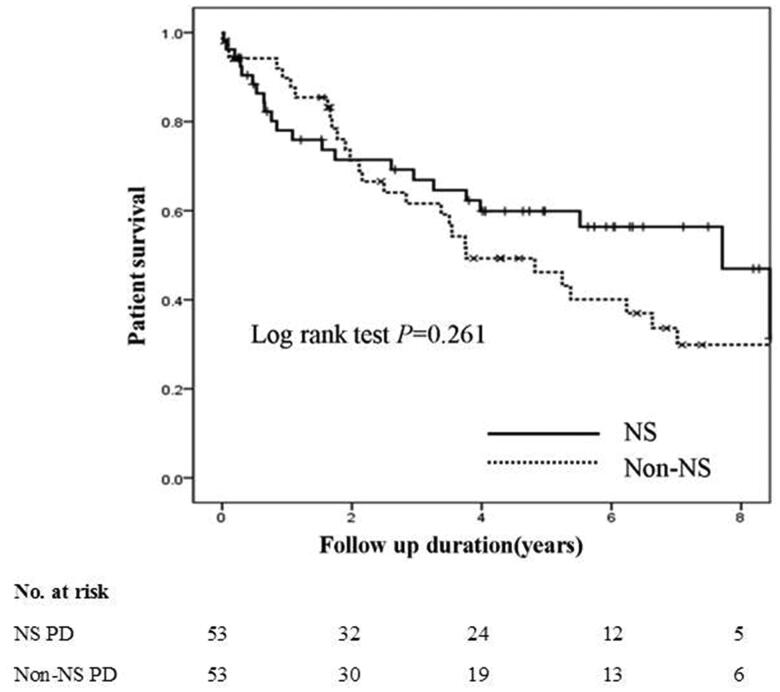
Kaplan–Meier’s overall survival curves of incident peritoneal dialysis patients with or without nephrotic syndrome.

Death occurred in 22 patients (41.5%) in the NS PD group and 31 patients (58.5%) in the non-NS PD group. The causes of death included cardiovascular disease (35.8%), peritonitis (9.4%), infection (13.2%), gastrointestinal disease (3.8%), malignancy (11.3%), multiple organ failure (5.7%), respiratory failure (7.5%), withdrawal (7.5%), and unknown (5.7%). There was no significant difference in the causes of death between the two groups (*p*= .591; Table 2). Surprisingly, the median survival time of patients in the NS PD group (6.60 years; 95% CI, 4.95–8.25 years) was similar to that of patients in the non-NS PD group (5.20 years; 95% CI, 4.05–6.34 years; *p*= .261) (Figure 2).

**Table 2.** Causes of death between PD patients with and without NS.

Causes of death	Total (*n* = 53)	NS PD group (*n* = 22)	Non-NS PD group (*n* = 31)
Cardiovascular disease	19 (35.8%)	7 (31.8%)	12 (38.7%)
Peritonitis	5 (9.4%)	2 (9.1%)	3 (9.7%)
Infection	7 (13.2%)	2 (9.1%)	5 (16.1%)
Gastrointestinal disease	2 (3.8%)	0 (0.0%)	2 (6.5%)
Malignancy	6 (11.3%)	2 (9.1%)	4 (12.9%)
Withdrawal	4 (7.5%)	2 (9.1%)	2 (6.5%)
Multiple organ failure	3 (5.7%)	2 (9.1%)	1 (3.2%)
Unknown	3 (5.7%)	2 (9.1%)	1 (3.2%)
Respiratory failure	4 (7.5%)	3 (13.6%)	1 (3.2%)

NS: nephrotic syndrome; PD: peritoneal dialysis.

The difference between the two groups was not significant (*p*= .591).

An interaction effect was observed between survival time and baseline NS status. Thus, patients’ mortalities within 1.5 years and after 1.5 years were analyzed separately.

### All-cause mortality within 1.5 years in incident PD patients with or without NS

**Figure 3.**
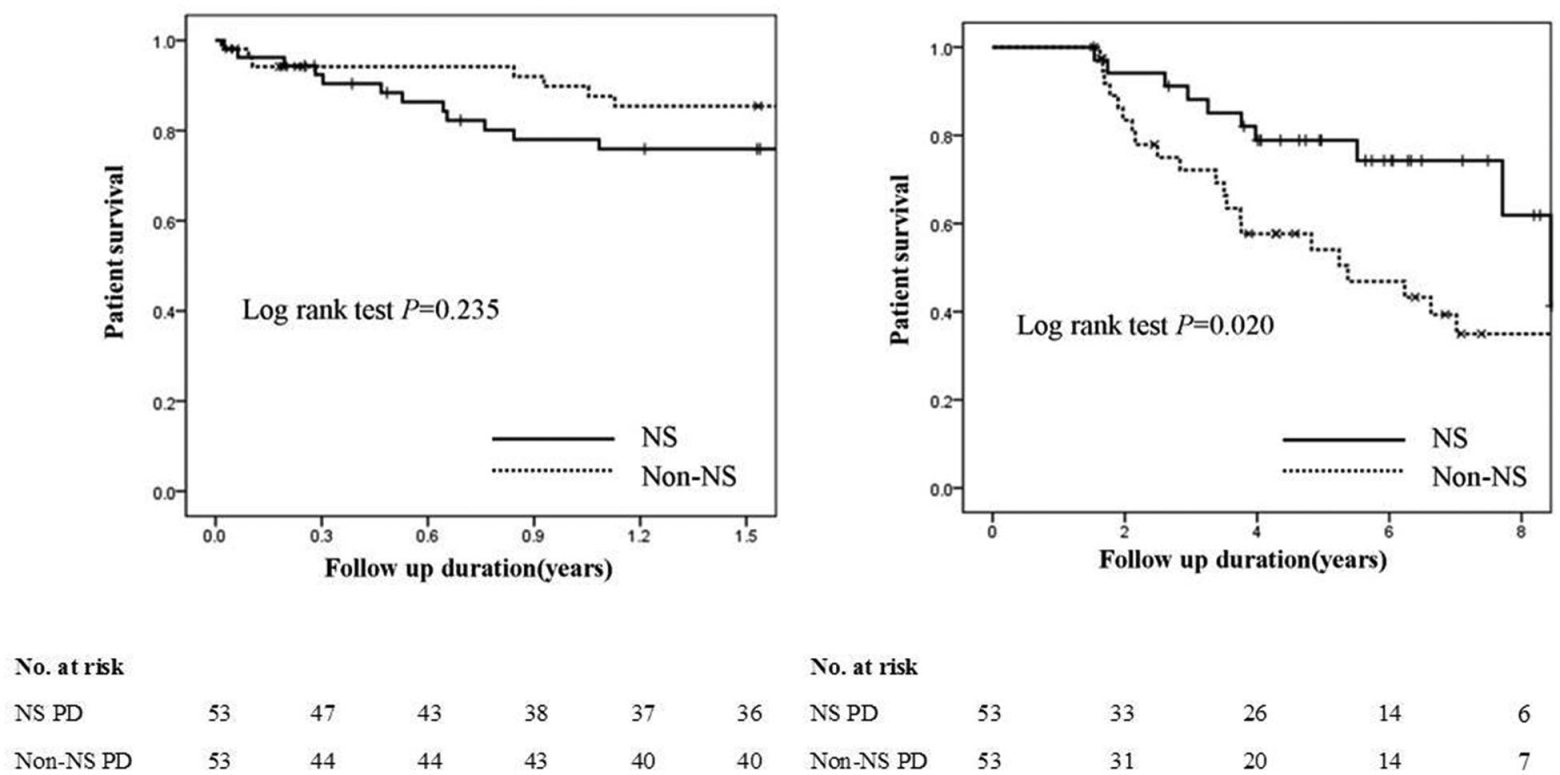
Kaplan–Meier’s survival curves within and after 1.5 years in incident peritoneal dialysis patients with or without nephrotic syndrome.

The mortality rate within 1.5 years in the patients with NS was comparable to that of the non-NS group (log-rank test, *p*= .235) (Figure 3). The multivariable Cox regression analysis showed that NS status (*p*= .387) at baseline was not associated with 1.5-year all-cause mortality on PD adjusting for BMI (HR, 0.84; 95% CI, 0.71–0.98; *p*= .031), serum creatinine (HR, 0.997; 95% CI, 0.994–1.000; *p*= .028), serum albumin levels at baseline (HR, 0.77; 95% CI, 0.61–0.96; *p*= .022), age, and hypertension (Table 3).

**Table 3.** Cox proportional hazard model for all-cause mortality of incident peritoneal dialysis patients with or without nephrotic syndrome.

Variable	Within 1.5 years				After 1.5 years			
	Univariate Cox regression		Multivariate Cox regression		Univariate Cox regression		Multivariate Cox regression	
	HR (95% CI)	*p-* value	HR (95% CI)	*p-* value	HR (95% CI)	*p-* value	HR (95% CI)	*p-* value
NS vs. non-NS	1.75 (0.69–4.44)	.241		.387	0.42 (0.20–0.90)	.024	0.38 (0.17–0.86)	.019
CHD	0.36 (0.05–2.70)	.321			1.62 (0.70–3.74)	.262		
Diabetes	0.87 (0.35–2.16)	.762			1.57 (0.76–3.23)	.225		
Stroke	1.14 (0.33–3.92)	.832			1.85 (0.83–4.15)	.134		
Hypertension	0.30 (0.09–1.02)	.054			0.30 (0.07–1.30)	.109		
Age (per 1-year increase)	1.05 (1.01–1.08)	.016			1.06 (1.03–1.09)	<.001	1.05 (1.02–1.08)	.001
Gender (female)	1.96 (0.77–4.97)	.159			1.01 (0.44–2.33)	.978		
BMI (per 1-kg/m^2^ increase)	0.86 (0.73–1.02)	.078	0.84 (0.71–0.98)	.031	1.01 (0.91–1.12)	.886		
eGFR (per 1-mL/min.1.73 m^2^ increase)	1.03 (0.94–1.12)	.544			1.02 (0.95–1.10)	.555		
Serum creatinine (per 1-μmol/L increase)	0.998 (0.996–1.000)	.051	0.997 (0.994–1.000)	.028	1.000 (0.998–1.001)	.385		
Serum urea (per 1-mmol/L increase)	0.97 (0.92–1.02)	.257			0.98 (0.94–1.02)	.360		
Serum albumin (per 1-g/L increase)	0.87 (0.77–0.99)	.035	0.77 (0.61–0.96)	.022	0.88 (0.78–1.00)	.043	0.86 (0.75–1.00)	.047
Hemoglobin (per 1-g/L increase)	1.00 (0.98–1.03)	.844			1.01 (0.99–1.02)	.422		
Serum calcium (per 1-mmol/L increase)	1.12 (0.23–5.36)	.889			1.65 (0.53–5.17)	.391		
Serum phosphorus (per 1-mmol/L increase)	0.66 (0.27–1.59)	.356			0.64 (0.31–1.34)	.239		
PTH (per 1-pg/mL increase)	0.998 (0.995–1.001)	.290			1.000 (0.999–1.001)	.665		

NS: nephrotic syndrome; BMI: body mass index; CHD: coronary heart disease; eGFR: estimated glomerular filtration rate; PTH: parathyroid hormone.

Multivariate Cox proportional hazard model included all the significant variables (*p*< .1) from the univariate analysis and baseline NS status. Variables in the final model were selected in a backward (LR) manner.

### All-cause mortality after 1.5 years in incident PD patients with or without NS

Compared with patients without NS, those with NS status at baseline had lower all-cause mortality after 1.5 years on PD (log-rank test, *p*= .020) (Figure 3). The multivariable Cox regression analysis showed that compared with patients without NS, PD patients with NS status (HR, 0.38; 95% CI, 0.17–0.86; *p*= .019) were significantly associated with lower all-cause mortality after 1.5 years on PD adjusting for age (HR, 1.05; 95% CI, 1.02–1.08; *p*= .001) and serum albumin levels at baseline (HR, 0.86; 95% CI, 0.75–1.00; *p*= .047) (Table 3).

### Technique failure of PD patients

**Figure 4.**
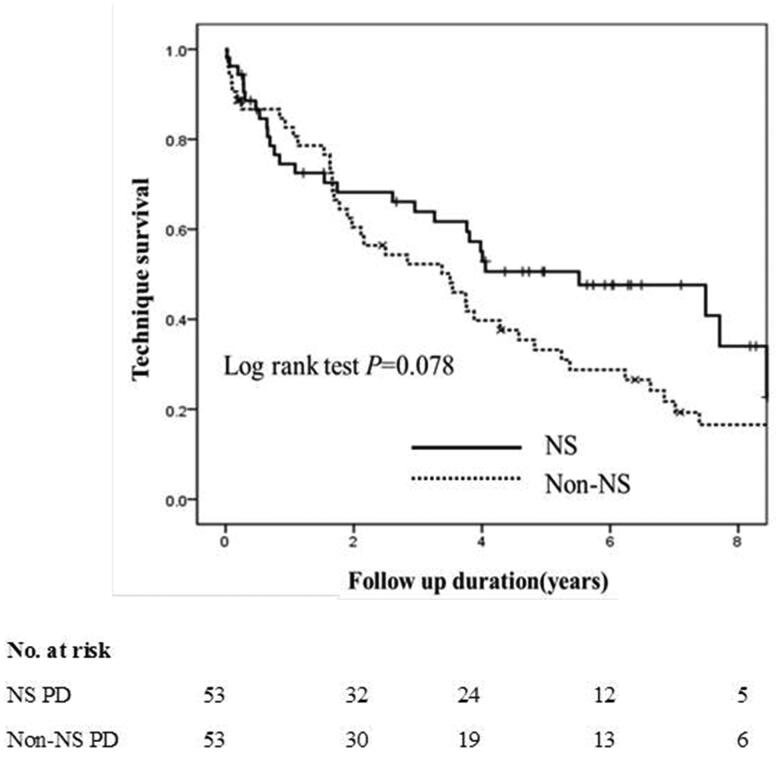
Kaplan–Meier’s overall technique survival curves of incident peritoneal dialysis patients with or without nephrotic syndrome.

Technique failure occurred in 28 patients (52.8%) in the NS PD group and 43 patients (81.1%) in the non-NS PD group. Six patients (11.3%) in the NS PD group and 12 patients (22.6%) in the non-NS PD group transferred to HD. Death occurred in 22 patients (41.5%) in the NS PD group and 31 patients (58.5%) in the non-NS PD group. The median technique survival time of patients in the NS PD group (5.73 years; 95% CI, 4.27–7.18 years) tends to be longer than that of patients in the non-NS PD group (4.11 years; 95% CI, 3.15–5.06 years, *p*= .078) (Figure 4).

An interaction effect was observed between technique survival time and baseline NS status. Thus, patients’ technique failure rates within 1.5 years and after 1.5 years were analyzed separately.

### Technique failure within 1.5 years in incident PD patients with or without NS

There were 2 and 4 patients who transferred from PD to HD within the 1.5-year follow-up in the NS and non-NS PD groups, respectively. The reasons of the two patients in the NS PD group were catheter complications and refractory peritonitis, respectively. In the non-NS PD group, the reasons were hydrothorax (one patient), catheter dislocation (two patients), and refractory peritonitis (one patient).

**Figure 5.**
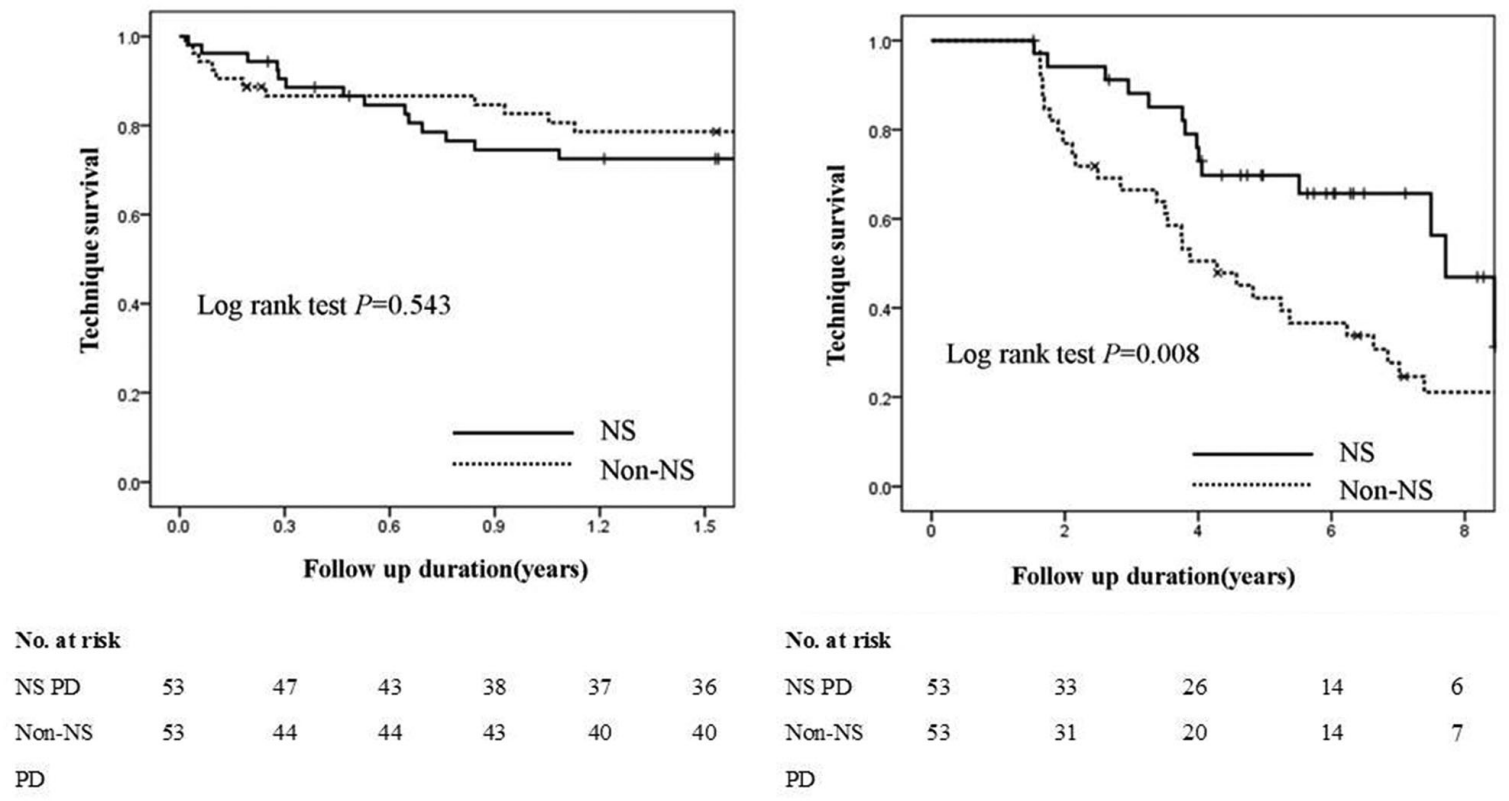
Kaplan–Meier’s technique survival curves within and after 1.5 years in incident peritoneal dialysis patients with or without nephrotic syndrome.

The technique failure rate in the patients with NS was comparable to that of the non-NS group (log-rank test, *p* = .543) (Figure 5). The multivariable Cox regression analysis showed that compared with patients without NS, those with NS status at baseline were not associated with 1.5-year technique failure on PD after adjusting for female sex (HR, 3.24; 95% CI, 1.28–8.16; *p*= .013), BMI, and serum albumin (Table 4).

**Table 4.** Cox proportional hazard model for technique failure of incident peritoneal dialysis patients with or without nephrotic syndrome.

Variable	Within 1.5 years				After 1.5 years			
	Univariate Cox regression		Multivariate Cox regression		Univariate Cox regression		Multivariate Cox regression	
	HR (95% CI)	*p-* value	HR (95% CI)	*p-* value	HR (95% CI)	*p-*value	HR (95% CI)	*p-* value
NS vs. non-NS	1.28 (0.58–2.82)	.544		.779	0.43 (0.23–0.82)	.010	0.45 (0.24–0.85)	.014
CHD	0.27 (0.04–2.02)	.204			1.35 (0.62–2.91)	.448		
Diabetes	0.95 (0.43–2.11)	.896			1.16 (0.64–2.09)	.636		
Stroke	1.15 (0.40–3.36)	.794			1.40 (0.67–2.93)	.370		
Hypertension	0.41 (0.12–1.37)	.148			0.29 (0.09–0.96)	.043		
Age (per 1-year increase)	1.02 (1.00–1.05)	.104			1.03 (1.01–1.05)	.013	1.03 (1.00–1.05)	.020
Gender (female)	2.18 (0.98–4.87)	.056	3.24 (1.28–8.16)	.013	1.08 (0.53–2.18)	.836		
BMI (per 1-kg/m^2^ increase)	0.89 (0.78–1.02)	.086			0.98 (0.89–1.08)	.662		
eGFR (per 1-mL/min.1.73 m^2^ increase)	1.05 (0.98–1.12)	.158			1.01 (0.95–1.08)	.661		
Serum creatinine (per 1-μmol/L increase)	0.999 (0.998–1.000)	.161			1.000 (0.999–1.001)	.863		
Serum urea (per 1-mmol/L increase)	0.98 (0.93–1.03)	.393			0.99 (0.96–1.03)	.731		
Albumin (per 1-g/L increase)	0.89 (0.80–1.00)	.055			0.94 (0.84–1.05)	.295		
Hemoglobin (per 1-g/L increase)	1.01 (0.99–1.03)	.636			1.01 (0.99–1.02)	.409		
Serum calcium (per 1-mmol/L increase)	2.06 (0.58–7.24)	.263			1.65 (0.62–4.43)	.320		
Serum phosphorus (per1-mmol/L increase)	0.85 (0.42–1.75)	.667			1.06 (0.61–1.85)	.840		
PTH (per1-pg/mL increase)	0.998 (0.996–1.001)	.229			1.000 (0.999–1.001)	.493		

NS: nephrotic syndrome; BMI: body mass index; CHD: coronary heart disease; eGFR: estimated glomerular filtration rate; PTH: parathyroid hormone.

Multivariate Cox proportional hazard model included all the significant variables (*p* < .1) from the univariate analysis and baseline NS status. Variables in the final model were selected in a backward (LR) manner.

### Technique failure after 1.5 years in incident PD patients with or without NS

After the 1.5-year follow-up, there were 4 and 8 patients who transferred from PD to HD in the NS and non-NS PD groups, respectively. The transferring reasons of the four patients with NS were catheter complications, refractory peritonitis, undergoing abdominal surgery, and inadequate dialysis. The reasons of the eight patients in the non-NS PD group were catheter complications (two patients), refractory peritonitis (four patients), and inadequate dialysis (two patients).

Compared with patients without NS, those with NS status at baseline were significantly associated with lower technique failure rate after 1.5 years on PD (log-rank test, *p*= .008) (Figure 5). The multivariable Cox regression analysis showed that compared with patients without NS, those with NS status at baseline (HR, 0.45; 95% CI, 0.24–0.85; *p*= .014) were significantly associated with lower risk of technique failure after 1.5 years on PD after adjusting for age (HR, 1.03; 95% CI, 1.00–1.05; *p*= .020) and hypertension (Table 4).

## Discussion

In the present study, we found that in patients with ESRD and NS treated with PD, both mortality and technique failure rates were not inferior to those of patients without NS within 1.5 years. Moreover, after 1.5 years of PD, patients with baseline NS had even better outcomes compared with those without NS.

For patients with NS, PD tends to be avoided due to the potential adverse effect of loss of protein from the peritoneum. Concerns are always there that loss of protein from drainage and urine could lead to the more severe hypoalbuminemia and malnutrition [12,13]. However, both short- and long-term outcomes of patients with NS treated with PD had not been fully elucidated.

Previous studies that investigated the effect of PD treatment for NS are generally scarce. Most studies are case reports and mainly focused on short-term PD treatment for refractory edema and fluid overload in patients with severe steroid-resistant NS. In those cases, short-term PD treatment that could provide ultrafiltration (UF) via the peritoneum was used as an effective alternative therapy for extracorporeal UF in both children [6] and adults [10,11, 14]. Barman et al. reported a successful treatment of edema using short-term PD in a child with diuretic-resistant NS and acute kidney injury (AKI). Significant improvement of blood pressure control, response to diuretic, and recovery from the AKI were achieved, and the procedure was tolerated well. The novel short-term use of PD was also mentioned by Harshman et al. [15] in an infant with congenital NS. Although fluid management was improved in that case, the infant died after 2 months of therapy because of multiple complications. In adult patients with NS, Takada et al. [14] introduced icodextrin-single PD therapy to a patient with idiopathic membranous nephropathy patient and overhydration. Refractory subcutaneous edema was alleviated, and remission of NS occurred after 2 weeks of PD treatment. The long-term outcomes of patients with NS were only mentioned in a 5-year retrospective case note review conducted by Dufek et al., who focused on infants with congenital nephrotic syndrome (CNS) [9]. In that study, chronic dialysis was commenced in 44 infants with CNS, while PD was the modality of choice in 93%. The complication, growth, and transplantation rates in infants with CNS on dialysis are comparable to those reported in infants with other primary renal diseases.

To our knowledge, our study is the first study that observed the outcomes of patients with ESRD and NS treated with PD. Most importantly, we compared the outcomes of these patients with those of the matched controls without NS using PSs based on age, sex, diabetes mellitus, and serum albumin. We found that both 1.5-year all-cause mortality and technique failure rates of patients in the NS PD group were not inferior to those in the non-NS PD group. Surprisingly, both the overall survival and technique survival of patients with NS after 1.5 years on PD were even better than those of the matched controls in the non-NS PD group. However, it should be noted that although the serum albumin levels of patients in the non-NS PD group were higher than those in the NS PD group, these patients had relatively lower serum albumin level. As lower serum albumin level is an important indicator for malnutrition, inflammation, and overhydration [16–18], both overall and technique survival disadvantages in the non-NS PD group may be due to these poor clinical conditions. Our results should be interpreted with caution since these are from the data in only one center. In spite of this, our results still provide some clue that with appropriate management, the outcomes of patients with ESRD and NS treated with PD were not inferior to those of matched control patients without NS.

The strengths of this study include its long-term follow-up, more precise match of patients with and without NS, and use of multivariable analyses to adjust for potentially confounding factors. Weighed against these strengths, the study had several limitations. First, patients included in this study were recruited from a single tertiary academic hospital in China, thereby raising the possibility of ascertainment bias. Second, due to the retrospective design, some detailed management information could not be obtained, such as detailed PD treatment regimen and, detailed dialysis adequacy and fluid removal as well as the absence of a quantification of diuresis. Finally, although we attempted to adjust for a range of demographic, clinical, and laboratory characteristics, some recently reported risk factors (such as serum alkaline phosphatase [19] and serum bilirubin levels [20]) were not included in our study; thus, residual confounding factors remain possible.

In conclusion, our study demonstrated that both short- and long-term PD outcomes in patients with ESRD and NS were not inferior to their matched control patients without NS, which indicated that PD could be considered as a long-term renal replacement therapy for patients with ESRD and NS.

## Data Availability

The datasets used and/or analyzed during the current study are available from the corresponding author on reasonable request.
